# On the Management of Convalescence from Acute Diseases

**Published:** 1847-05

**Authors:** 


*On the Management of Convalescence from Acute Disease.* By M. Reveille Parise.—M. Parise remarks that after serious prolonged illness every organ suffers more or less from exhaustion, and its function is feebly performed. This condition of the economy results from the violent excitement of the antecedent disease and the privation of food. The former, of course, no longer operates, and food may be given to replenish the waste, and recruit the forces, the stomach is therefore the organ with which we have to do.

M. Parise lays it down as a maxim that, after disease, the sensibility of the stomach and intestines is exalted and their tone diminished. The indications therefore are obviously to diminish the sensibility and to augment the tone of the stomach, and consequently to enable digestion to be properly performed; but our means of accomplishing this are not so efficacious as might be imagined. If the subject be young and vigorous, health is soon restored, and digestion established; but even here, if the tormenting hunger which accompanies convalescence be appeased by injudicious supplies of food, various disturbances of the digestive organs ensue. 'But if the convalescent is naturally delicate, nervous, or irritable, if his digestive powers are not strong even in health, if he has reached a certain age, or been worn by anxiety, we must expect a tedious convalescence."

At first sight, the fortifying the stomach by tonics would seem to be a natural means of procedure; but every reflecting practitioner has seen cases in which an irritable and sensitive condition of the stomach renders the establishment of its powers a matter of nicety, as well as difficulty. If we employ a soothing means and a debilitating regimen, the tone of the organ is further diminished; while if we exhibit stimulants, uneasiness and thirst, dryness of the mouth, &c., prove that we are injuriously exciting the organ. Diarrhœa occurring during convalescence often misleads the practitioner, for he has difficulty in determining whether it is caused by some source of irritation still remaining, or whether it is the consequence of simple atony of the intestinal canal. In these cases it may be stated as a general rule, that the diarrhœa of convalescence is connected with defective tonicity, which will be yet further diminished by abstinence and local depletion.

The following indications form the basis of a judicious convalescent regimen:

1. To allow only as much food as the stomach can digest.
2. To advise the patient to eat little and often.
3. To submit the food to effectual mastication.
4. To keep the general surface, and especially the extremities, warm during digestion.
5. To adapt the food to the peculiar sympathies of the stomach.
6. To introduce a judicious variety in the diet.
7. To advise change of air.
8. To avoid vivid moral emotions.

It is certain, notwithstanding, that however properly the above injunctions be carried out, the recovery may be impeded by various accidents. Only two of these are alluded to by M. Parise, and that because of their frequency, viz: *diarrhœa* and *gastro-enteralgia*. When the first of these is present we must endeavor to ascertain whether it is produced by errors of diet or by some moral cause. Is there any inflammation, or does it depend upon simple atony of fibre? Even when symptoms believed to be inflammatory are present, we must still be very cautious in recommending abstinence and leeching, which may produce a degree of exhaustion and loss of contractility of parts, which may take years for its reparation. Diminution of food will, however, be required, as well as counter-irritation, in the form of dry cupping, or sinapisms to the abdomen. When the irritation has subsided, mild tonics and an improved diet are indicated. In passive diarrhœa, M. Parise speaks of the *theriacum* (a miscellaneous compound containing opium) combined with calumba.

*Gastralgia*, or *enteralgia*, is far more frequent after severe disease, especially when the alimentary canal has been effected, than is generally believed. The irregularity forms one of its most dirtinguishing features. The acute feeling of hunger suddenly changes into a state of insupportable languor. In this state a superabundant nourishment is injurious, but in a far less degree than a too scanty one. Abstinence cannot be borne, nor do the epigastric pain and sinking cease until a certain quantity of food has been swallowed; while, if its administration be too long delayed, digestion does not take place, and diarrhœa is apt to ensue. First among medicines adapted to this state is bismuth alone, or with opium and calumba. Blisters and sinapisms to the epigastrium are also beneficial, as is also the endermic use of morphia.

Whatever means we have recourse to for the restoration of the energy of the alimentary canal, their prolonged employment is necessary. "Perseverance and variety," says M. Parise, "must never be lost sight of in a troublesome convalescence, during which health and disease are constantly vibrating."—*Medico-Chirurg. Rev.—Ibid.*

